# *Angiopteris guangdongensis* (Marattiaceae): A New Species From Guangdong, China

**DOI:** 10.1002/ece3.72447

**Published:** 2025-11-09

**Authors:** Wu‐Feng Chen, Wei‐Yue Sun, Li‐Jun Chen, Jiang‐Ping Shu, Jun‐Jie Liang, Yue‐Bing Zheng, Yue‐Hong Yan

**Affiliations:** ^1^ School of Life and Health Sciences Hunan University of Science and Technology Xiangtan China; ^2^ Key Laboratory of National Forestry and Grassland Administration for Orchid Conservation and Utilization The Orchid Conservation & Research Center of Shenzhen Shenzhen China; ^3^ Shanghai Chenshan Botanical Garden, Chenshan Scientific Research Center of CAS Center for Excellence in Molecular Plant Sciences Shanghai China; ^4^ Shenzhen Key Laboratory of Southern Subtropical Plant Diversity, Fairy Lake Botanical Garden Shenzhen & Chinese Academy of Sciences Shenzhen China; ^5^ Management Office, Guangdong Enping Qixingkeng Provincial Nature Reserve Enping China

**Keywords:** ferns, Marattiaceae, morphology, phylogeny, plastid genome, taxonomy

## Abstract

In this study, we confirm that the population of *Angiopteris* from Guangdong Province represents a distinct new species, which we describe as *Angiopteris guangdongensis*. Morphologically, *A. guangdongensis* resembles *Angiopteris fokiensis* but can be distinguished by its arborescent habit (reaching up to 5 m in height), robust scaly stipes, linear‐lanceolate pinnules, abaxially scaly slender pinnules, sori with 7–16 sporangia, and a higher basal pinnule aspect ratio. Plastid phylogenomic analyses place *A. guangdongensis* as a monophyletic lineage sister to *A. fokiensis* . According to IUCN guidelines, the species is preliminarily assessed as ‘Data Deficient (DD)’. In addition, we report the complete plastid genome of this new species. This discovery not only provides important insights into the evolution and speciation of *Angiopteris* but also highlights the taxonomic oversimplification within the genus.

## Graphical abstract

In this study, we confirm that the population from Guangdong Province represents a distinct new species, which we describe here as Angiopteris guangdongensis. Morphologically, *A. guangdongensis* is similar to Angiopteris fokiensis but distinguished by its arborescent habit, robust stipes, pinnules linear‐lanceolate, slender pinnules, number of sporangia and basal pinnule aspect ratio. Plastid phylogenomic analyses resolve *A. guangdongensis* as a monophyletic lineage sister to *A. fokiensis* .

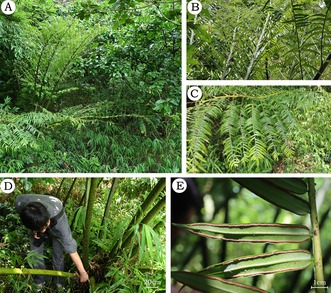

## 1 Introduction

The genus *Angiopteris* Hoffm. (1796, 29), belonging to the family Marattiaceae Kaulf. (1824, 31), represents an evolutionarily significant group of ancient giant ferns widely distributed across the Paleotropics (Murdock 2008; He and Christenhusz 2013). Despite its importance, the taxonomy of the genus remains problematic. Previous phylogenetic analyses based on chloroplast DNA fragments have failed to resolve well‐supported relationships, particularly for key species such as *Angiopteris evecta* (G. Forst.) Hoffm. (1794, 29) and *Angiopteris fokiensis* Hieron. (1919: 275). Both exhibit polyphyletic patterns, suggesting the existence of multiple cryptic species (Murdock 2008). These challenges highlight the considerable difficulties faced in clarifying the taxonomy of this genus.

Current taxonomic treatments of *Angiopteris* reveal significant discrepancies in species recognition. Plants of the World Online (POWO 2023, https://powo.science.kew.org/) recognises approximately 58 species globally, whereas Flora of China lists only about 30 species, including 17 occurring in China (He and Christenhusz 2013). This contrasts sharply with Ching's earlier treatment, which documented 62 species of *Angiopteris* (including 43 newly described taxa) and 9 species of *Archangiopteris* in China (Ching 1959). Subsequent revisions (Flora of Fujian 1991; Lin 2006; He 2009) synonymised 14 of these species under *A. fokiensis* —including *Angiopteris muralis* Ching (1959, 343)—thereby creating a geographically widespread and potentially polyphyletic species complex. These extensive taxonomic modifications underscore ongoing issues of misidentification and problematic synonymisation within the genus, with most species remaining poorly studied (He and Christenhusz 2013; Zhou and He 2025).

With advances in molecular phylogenetic analyses, the relationships within the genus *Angiopteris* have been progressively revised and refined. Early revisions relied mainly on morphological data and limited chloroplast sequences, as exemplified by the taxonomic reassessment of *Angiopteris tonkinensis* by Wang et al. (2020). The subsequent adoption of plastid genome data provided higher resolution, enabling the recognition of species such as *Angiopteris nodosipetiolata* (Wang et al. 2024). More recently, Zhao et al. (2023) employed 26 transcriptomic datasets to reconstruct the phylogeny of the family Marattiaceae, establishing a robust phylogenomic framework for understanding the evolutionary history of marattioid ferns. Collectively, these studies have substantially advanced the taxonomy and systematics of this ancient lineage.

During recent field investigations in Qixingkeng Provincial Nature Reserve, Guangdong Province, we collected distinctive specimens of a giant marattioid fern with diagnostic morphological features. These include an arborescent habit, robust and densely scaly stipes, abaxially scaly slender pinnules, sori containing 7–16 sporangia, and a higher basal pinnule aspect ratio (AR), all of which distinguish it from *A. fokiensis* . Integrated molecular phylogenetic and morphological evidence confirms that these specimens represent a new, previously undescribed species, which we designate as *Angiopteris guangdongensis* Wufeng Chen & Y.H. Yan. Furthermore, by presenting the first complete plastid genome of a new *Angiopteris* species—sequenced from type‐locality samples of *A. fokiensis* and *A. muralis* —we provide precise baseline genomic data. This discovery not only offers critical insights into the evolution and speciation of *Angiopteris* but also underscores the prevailing taxonomic oversimplification within the genus.

## 2 Materials and Methods

### 2.1 Morphological Study

Plant materials of *Angiopteris* were collected from Qixingkeng Provincial Nature Reserve during field surveys conducted between 2023 and 2024. Voucher specimens (YYH24298.1, YYH24298.3–YYH24298.5) have been deposited at the National Orchid Conservation Center (NOCC) and the Chenshan Herbarium (CSH). Morphological characterisation was conducted using both dried specimens and field observations. Taxonomic comparisons with related species were based on examination of herbarium collections, critical review of relevant literature (e.g., *Flora of China*), and consultation of digital databases (IPNI, CVH, GBIF). In addition, comparative morphological analyses of *A. guangdongensis* , *A. muralis* and *A. fokiensis* were performed using an OLYMPUS‐SZ61 stereomicroscope and a JSM‐IT210LV scanning electron microscope (SEM) to identify diagnostic features.

### 2.2 Morphological Statistical Analysis

Specimens associated with the measurement data in this study are listed in Table 1. Statistical analyses were performed using GraphPad Prism v10.1.2 for Windows (GraphPad Software 2023). The normality of continuous variables was assessed using the Shapiro–Wilk test, complemented by visual inspection of Q–Q plots. When both normality and homogeneity of variance assumptions were met, parametric analysis was conducted using one‐way analysis of variance (ANOVA) followed by Tukey's post hoc test for planned group comparisons. When normality was satisfied but variances were unequal, robust ANOVA (Brown‐Forsythe or Welch) with Dunnett's T3 post hoc test was applied. For non‐normally distributed data, the Kruskal–Wallis test was used, followed by Dunn's test with Bonferroni correction for multiple comparisons. Results are reported as mean ± SD for normally distributed data or as median (interquartile range) for non‐normally distributed data. Statistical significance was set at *α* = 0.05, with exact *p*‐values provided.

**TABLE 1:** Specimens associated with the measurement data in this study.

No.	Specimen voucher (Herbaria)	Species name
1	YYH24298 (NOCC)	*Angiopteris guangdongensis* Wufeng Chen & Y.H. Yan
2	YYH24298.3 (NOCC)	*Angiopteris guangdongensis* Wufeng Chen & Y.H. Yan
3	YYH24298.4 (NOCC)	*Angiopteris guangdongensis* Wufeng Chen & Y.H. Yan
4	YYH24298.5 (NOCC)	*Angiopteris guangdongensis* Wufeng Chen & Y.H. Yan
5	YYH16796 (NOCC)	*Angiopteris fokiensis* Hieron.
6	YYH16797 (NOCC)	*Angiopteris fokiensis* Hieron.
7	PE00044427 (PE)	*Angiopteris fokiensis* Hieron.
8	PE00044428 (PE)	*Angiopteris fokiensis* Hieron.
9	YYH16795 (NOCC)	*Angiopteris fokiensis* Hieron.
10	PE00044459 (PE)	*Angiopteris fokiensis* Hieron.
11	YYH16801 (NOCC)	*Angiopteris fokiensis* Hieron.
12	YYH16802 (NOCC)	*Angiopteris fokiensis* Hieron.
13	BM000787027 (BM)	*Angiopteris fokiensis* Hieron.
14	YYH15501 (NOCC)	*Angiopteris muralis* Ching
15	YYH24821 (NOCC)	*Angiopteris muralis* Ching
16	YYH20553 (NOCC)	*Angiopteris muralis* Ching

### 2.3 Taxon Sampling, DNA Extraction and Sequencing

In this study, four new *Angiopteris* plastomes were sequenced and fully assembled. Detailed sample information is provided in Table S3. Fresh leaf material was silica‐dried prior to commercial sequencing. Total genomic DNA was extracted using the CTAB method, and short‐read sequencing (2 × 150 bp) was performed on the DNBSEQ‐T7RS platform by GrandOmics (Wuhan, China).

### 2.4 Plastome Assembly and Annotation

The raw sequencing data for each sample were quality‐checked using FastQC v0.11.9 (Andrews 2010) with default parameters. High‐quality paired‐end reads were assembled into contigs using GetOrganelle v1.7.7.1 (Jin et al. 2020), with parameters set to R (maximum extension rounds) = 10 and k (k‐mer sizes) = 21, 45, 65, 85, 105, 127. The plastome of *Angiopteris fokiensis* (NC_068854) served as the reference. Assembled plastomes were visualised in Bandage (Wick et al. 2015) to confirm circularisation, resulting in complete circular plastomes for all samples. Annotation was conducted using the PGA.pl. script from the plastid genome annotator (PGA) package (Qu et al. 2019) with the same reference (NC_068854), followed by manual curation in Geneious Prime v2024.0.5 (Kearse et al. 2012). The final annotated plastome maps were generated with OGDRAW (https://chlorobox.mpimp‐golm.mpg.de/OGDraw.html; Greiner et al. 2019). To compare chloroplast genome variation between *A. guangdongensis* and closely related species, global sequence alignment was conducted using the online software mVISTA (https://genome.lbl.gov/vista/index.shtml) (Brudno et al. 2003) in Shuffle‐LAGAN mode (Frazer et al. 2004), with *Angiopteris fokiensis* as the reference. The final dataset comprised 26 plastomes (Table S1) representing five major clades (Wang 2022) and key distribution regions, including Hainan, Guangxi, Guangdong, Yunnan, Fujian and Tibet, thereby providing a robust phylogenetic framework for comparative analysis of *Angiopteris*.

### 2.5 Phylogenetic Analyses

To elucidate phylogenetic relationships among the newly sequenced specimens, we conducted maximum likelihood (ML) and Bayesian inference (BI) analyses, using *Angiopteris latipinna* (YYH16216) as the outgroup. This species was historically classified under *Archangiopteris*, now synonymised with *Angiopteris* (He and Christenhusz 2013), and its position as a phylogenetically proximate sister lineage to the ingroup taxa makes it an appropriate root for topology reconstruction (Wang 2022). Sequence alignment and trimming of complete plastomes were performed using MAFFT v7.526 (Katoh and Standley 2013) and trimAl v1.5.0 (Capella‐Gutiérrez et al. 2009), respectively. Model selection was conducted in PhyloSuite using the ModelFinder function, with both the corrected Akaike Information Criterion (AICc) and Bayesian Information Criterion (BIC) supporting GTR + F + I as the best‐fit nucleotide substitution model (Kalyaanamoorthy et al. 2017). ML analysis was performed in IQ‐TREE v2.3.6 (Bui et al. 2020) with 1000 bootstrap replicates, while BI analysis was conducted using PhyloSuite v1.2.3 (Zhang et al. 2020). The BI workflow incorporated format conversion tools (Xiang et al. 2023), model selection (Kalyaanamoorthy et al. 2017), and MrBayes v3.2.6 (Ronquist et al. 2012), with runs conducted for 1000,000 generations. Convergence was considered achieved when the average standard deviation of split frequencies fell below 0.01, following the MrBayes manual (Ronquist et al. 2019). Final tree visualisation and annotation were generated using tvBOT (https://chiplot.online/tvbot.html; Xie et al. 2023).

## 3 Results

### 3.1 Plastome Characteristics of the Genus *Angiopteris*

**FIGURE 1:**
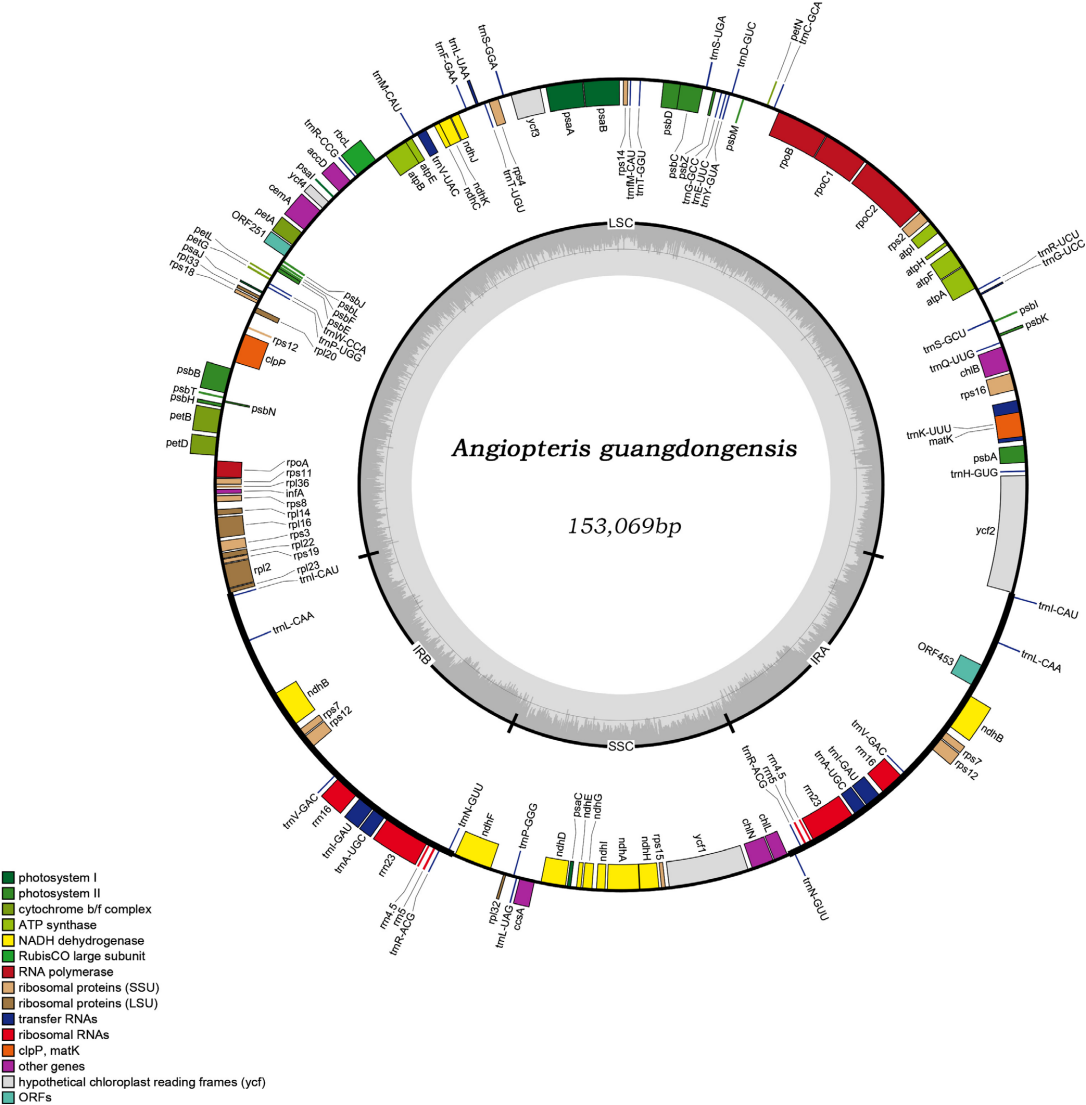
Chloroplast genome map of *Angiopteris guangdongensis*. Genes on the inner side of the circle are transcribed clockwise, whereas those on the outer side are transcribed counterclockwise. The dark grey inner circle represents GC content. Different colours indicate distinct functional gene categories. The thick line on the outer circle marks the boundaries of the IRA and IRB, separating the genome into SSC and LSC regions.

We assembled and annotated four new plastomes, which have been deposited in GenBase (Table S3). All four newly sequenced *Angiopteris* plastomes were 153,069 bp in length, comprising a Large Single‐Copy (LSC) region of 89,712 bp, a Small Single‐Copy (SSC) region of 20,585 bp and two inverted repeat regions of 21,386 bp each. The overall Guanine‐Cytosine (GC) content was the same across samples (35.40%). The plastome of *Angiopteris guangdongensis* contained 135 genes, including 88 protein‐coding genes, 39 tRNA genes and 8 rRNA genes (Table S3; Figure 1).

**FIGURE 2:**
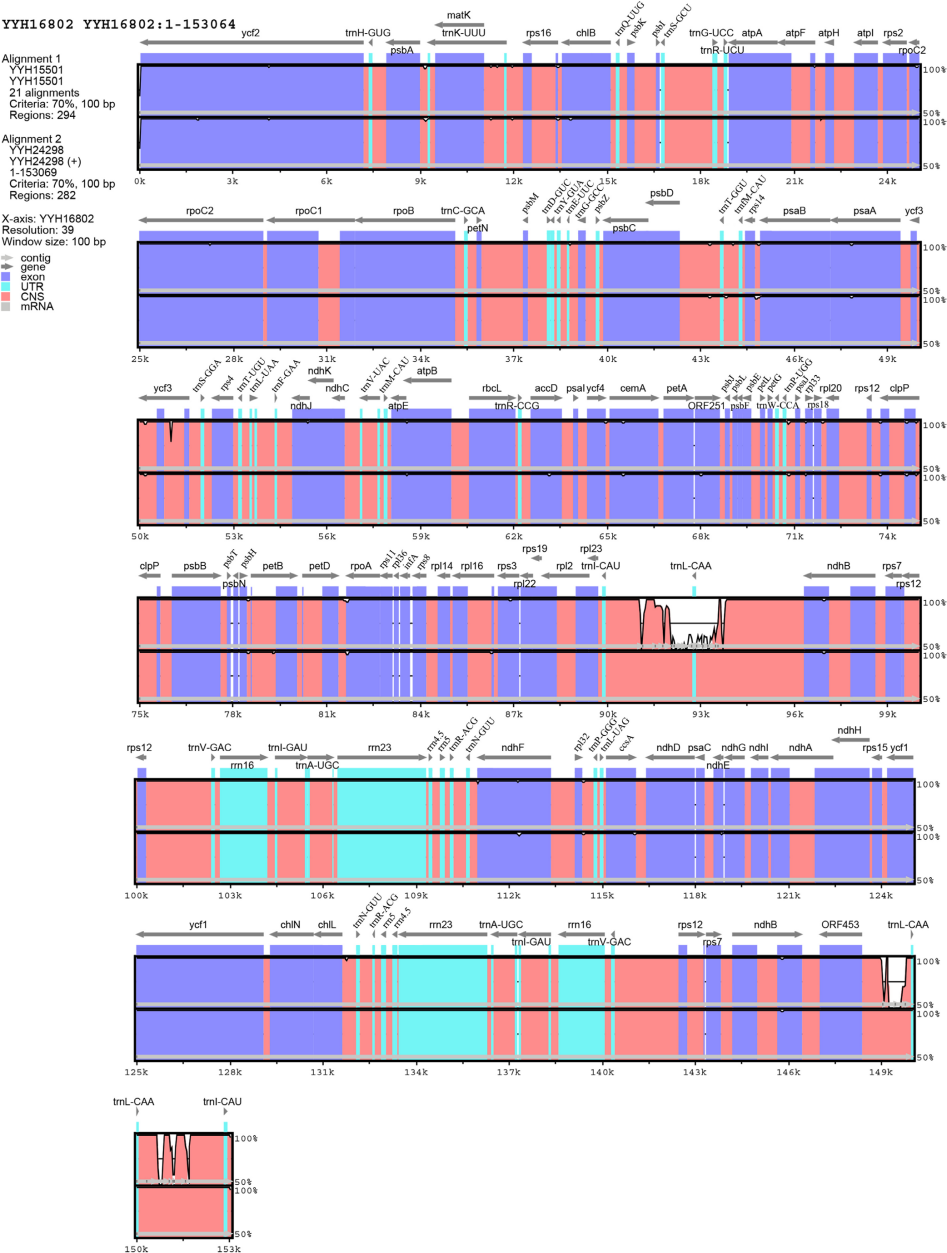
Global sequence alignment analysis of *A. guangdongensis* and closely related species. Grey arrows indicate gene transcription directions, purple regions represent coding sequences, red regions denote non‐coding sequences, cyan regions mark tRNA or rRNA. The horizontal axis shows alignment length (bp), while the vertical axis indicates sequence similarity (50%–100%). In the figure, YYH16802, YYH15501, and YYH24298 correspond to *A. fokiensis* , *A. muralis* , *A. guangdongensis* , respectively.

Global sequence alignment of *A. guangdongensis* and closely related species revealed overall conserved plastome architecture, with notable variation in the protein‐coding gene *ycf3* and the *trnI‐CAU–trnI‐CAU* spacer (Figure 2). Unlike *A. guangdongensis* and *A. fokiensis* , the *ycf3* gene in *A. muralis* is interrupted by a non‐coding sequence of approximately 3 kb. Furthermore, while the *trnI‐CAU–trnL‐CAA* spacer is highly similar between *A. guangdongensis* and *A. fokiensis* , it shows marked divergence in *A. muralis* .

### 3.2 Phylogenetic Relationships Within *Angiopteris*

**FIGURE 3:**
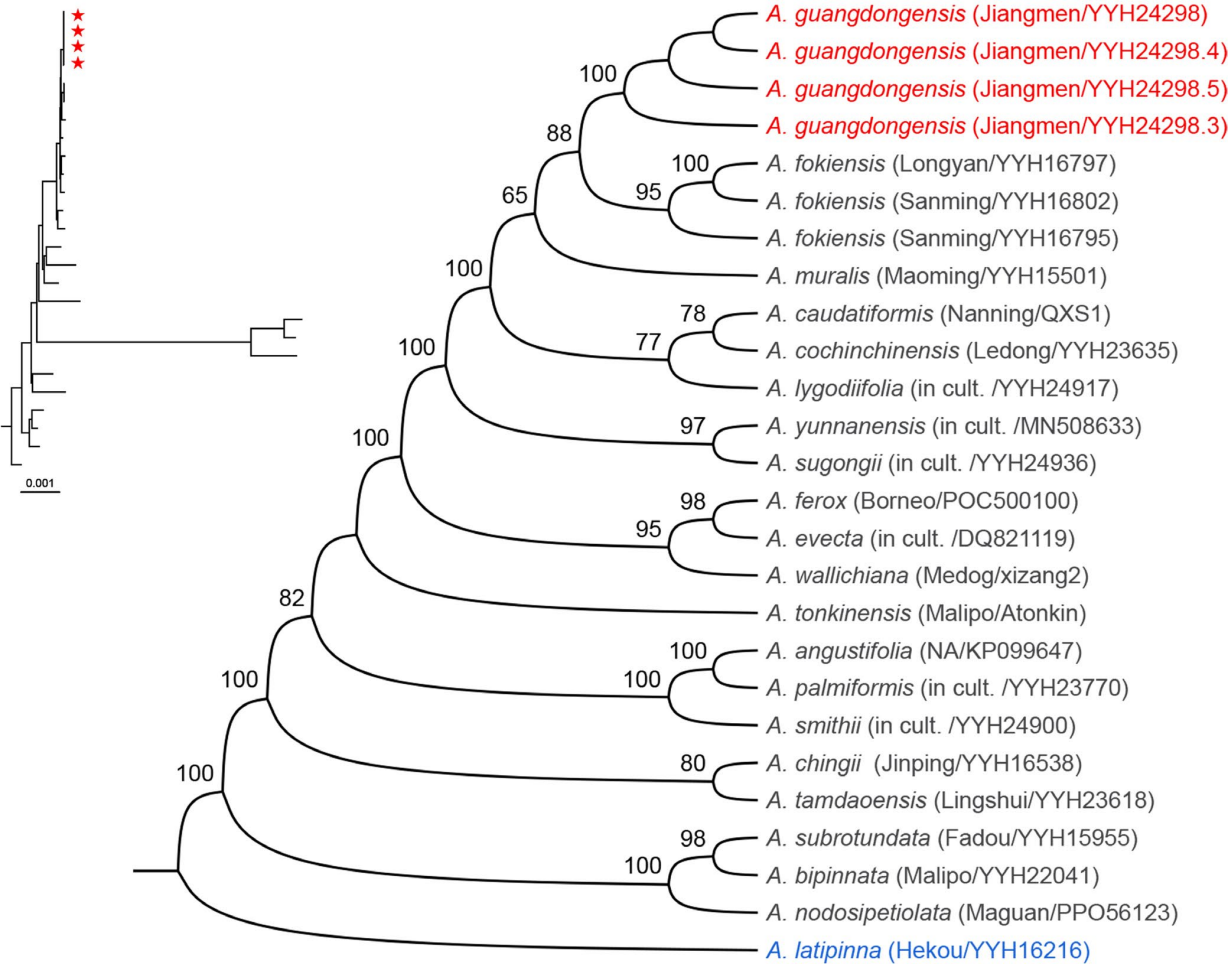
Maximum likelihood (ML) phylogenetic tree of *Angiopteris* based on 26 complete plastid genomes. Numbers at nodes indicate bootstrap support (BS) values (> 50%). Red stars or red text denote sequences of the new species, while outgroups are shown in blue text. Voucher information is provided in Table S1. ‘NA’ indicates data not available for sampling site information.

**FIGURE 4:**
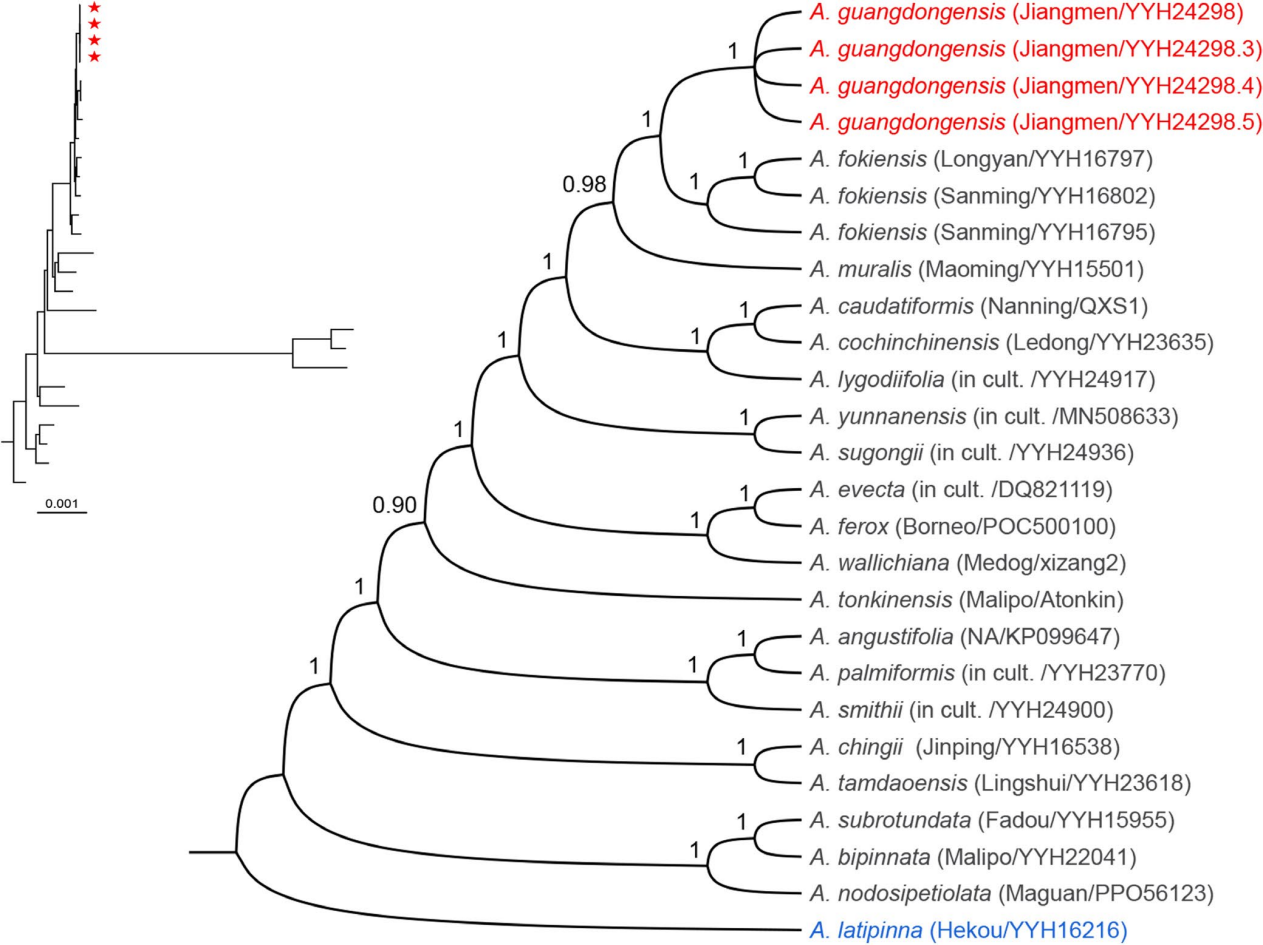
Bayesian Inference (BI) phylogenetic tree of *Angiopteris* based on 26 complete plastid genomes. Numbers at nodes indicate posterior probability (PP) values. Red stars or red text denote sequences of the new species, while outgroups are shown in blue text. Voucher information is provided in Table S1. ‘NA’ indicates data not available for sampling site information.

Phylogenetic analyses of 26 complete plastid genomes were conducted using *Angiopteris latipinna* (YYH16216) as the outgroup. Both ML (Figure 3) and BI (Figure 4) trees provided strong support for the monophyly of *A. guangdongensis* (bootstrap support [BS] = 100%, PP = 1), resolving it as sister to *A. fokiensis* . Furthermore, the analyses identified *A. muralis* as sister to the clade comprising *A. guangdongensis* and *A. fokiensis* . Nevertheless, *A. guangdongensis* exhibited a comparatively distant genetic relationship with *A. muralis* .

### 3.3 Taxonomic Treatment

**FIGURE 5:**
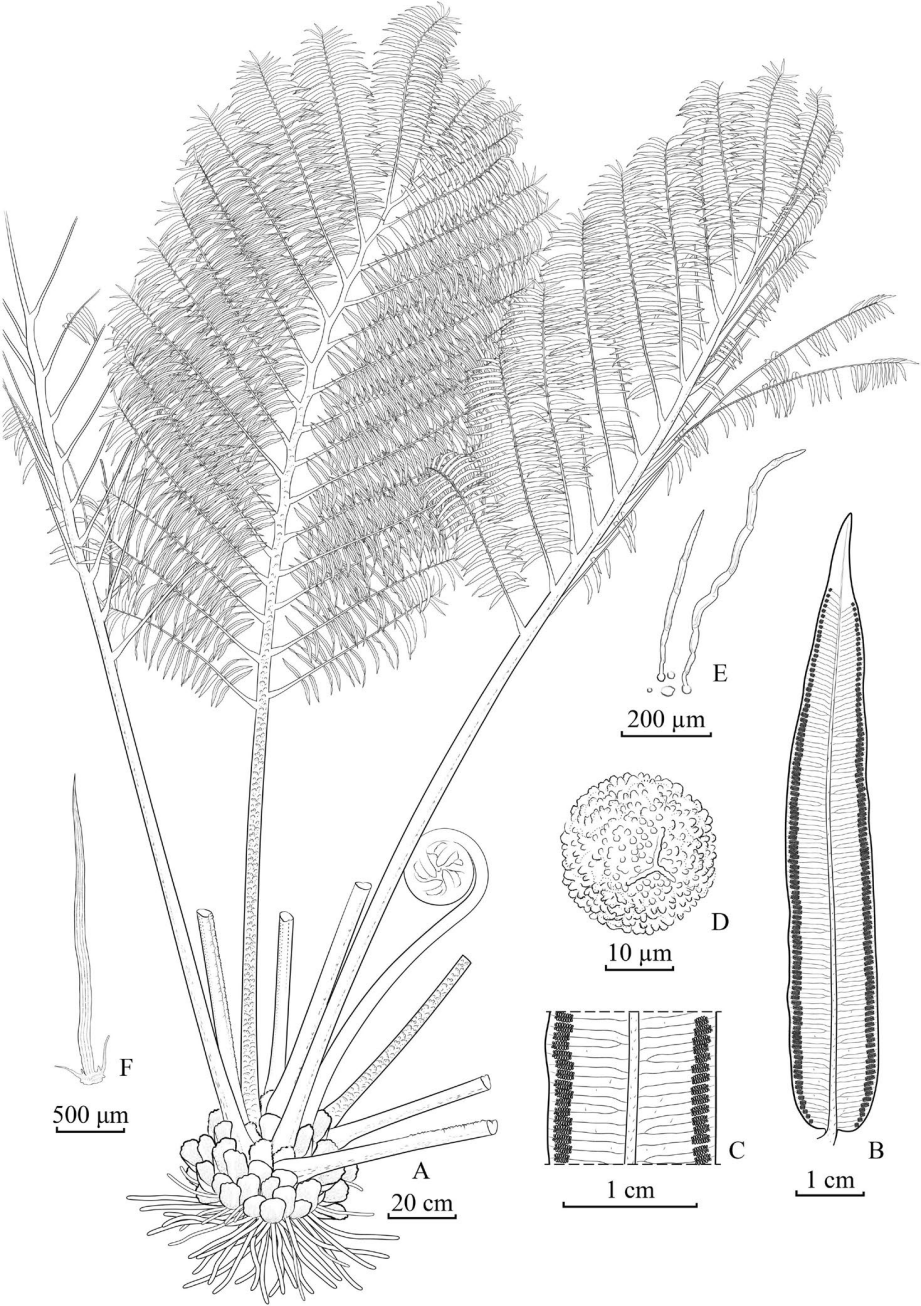
*Angiopteris guangdongensis* Wufeng Chen and Y.H. Yan, sp. *nov*. (A) Habit; (B) pinnule; (C) detail of pinnule; (D) spore; (E) scale on the abaxial surface of the pinnule; (F) scale on the abaxial surface of the pinna rachis.

**FIGURE 6:**
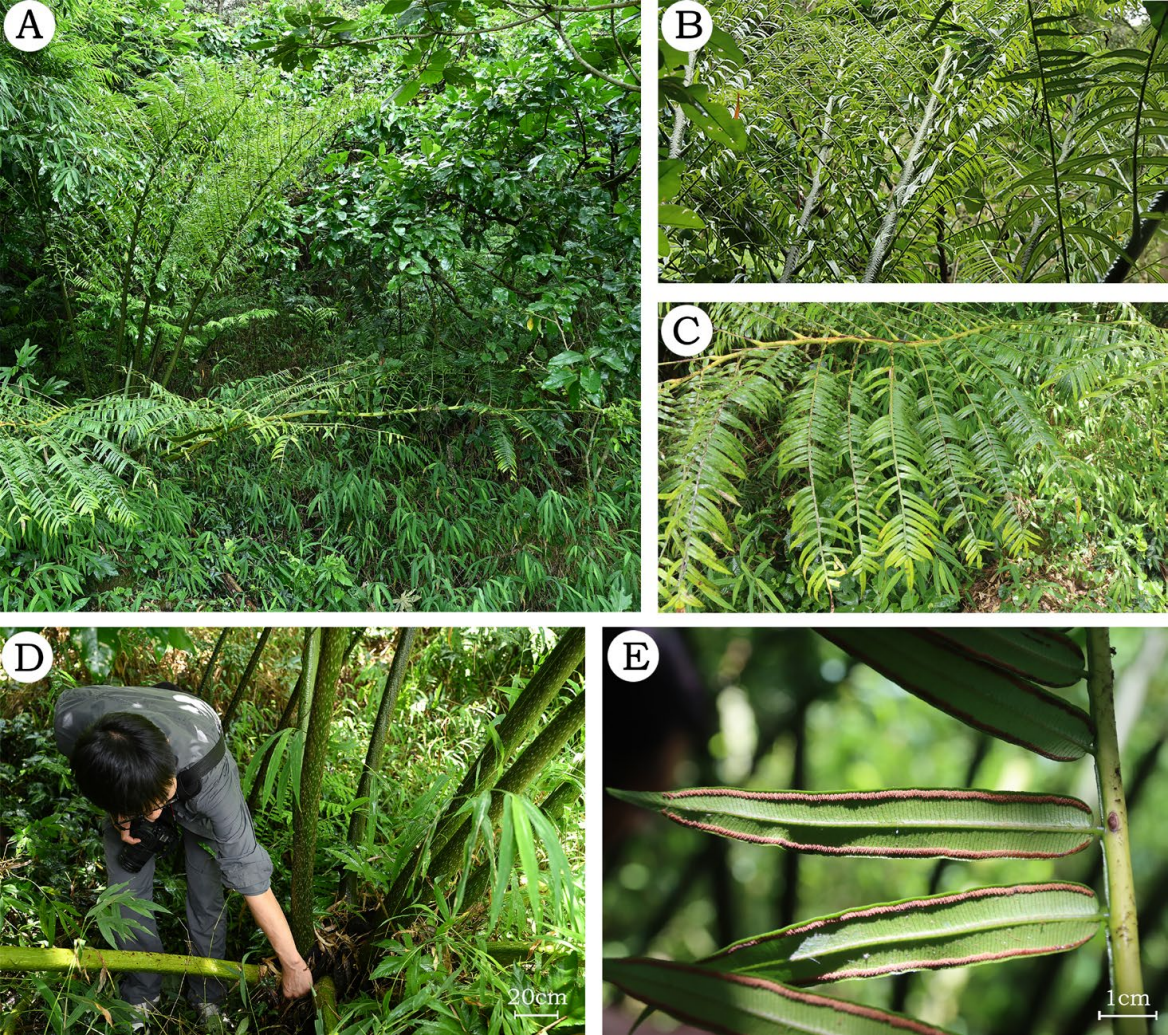
*Angiopteris guangdongensis* Wufeng Chen and Y.H. Yan, sp. *nov*. (A) Habitat; (B) lamina; (C) pinnae; (D) rhizome and stipe; (E) sporangia. Photographs by Jin‐Gang Liu (A–E).

**FIGURE 7:**
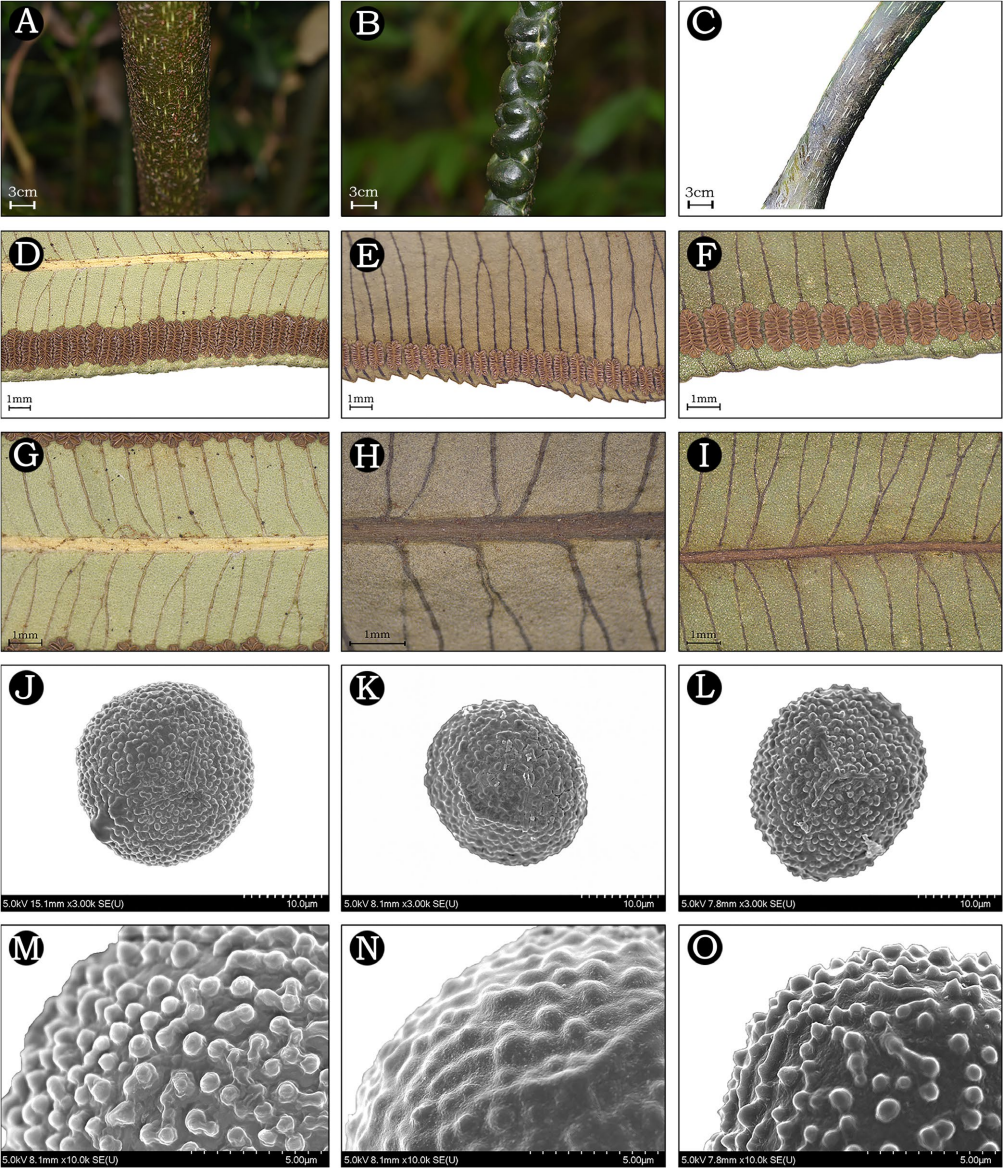
Comparison of the stipe and pinnule of *A. guangdongensis* (left), *A. muralis* (middle) and *A. fokiensis* (right). (A–C) Stipe; (D–F) veins and sori; (G–I) scales along the midrib; (J–L) tetrahedral spores; (M–O) exospores with verrucate ornamentation. Photographs by Chao‐Qi Wang (J–O).

***Angiopteris guangdongensis*** Wufeng Chen and Y.H. Yan, sp. *nov*. (Figures 5, 6, 7)

**Type**: CHINA. Guangdong Province: Maoming City, Xinyi County, 22**°**10**′** N, 112**°**6′ E, elev. 149–165 m, 29 May 2023, Ting Wang YYH24298 (holotype: NOCC!; isotype: CSH!)

**FIGURE 8:**
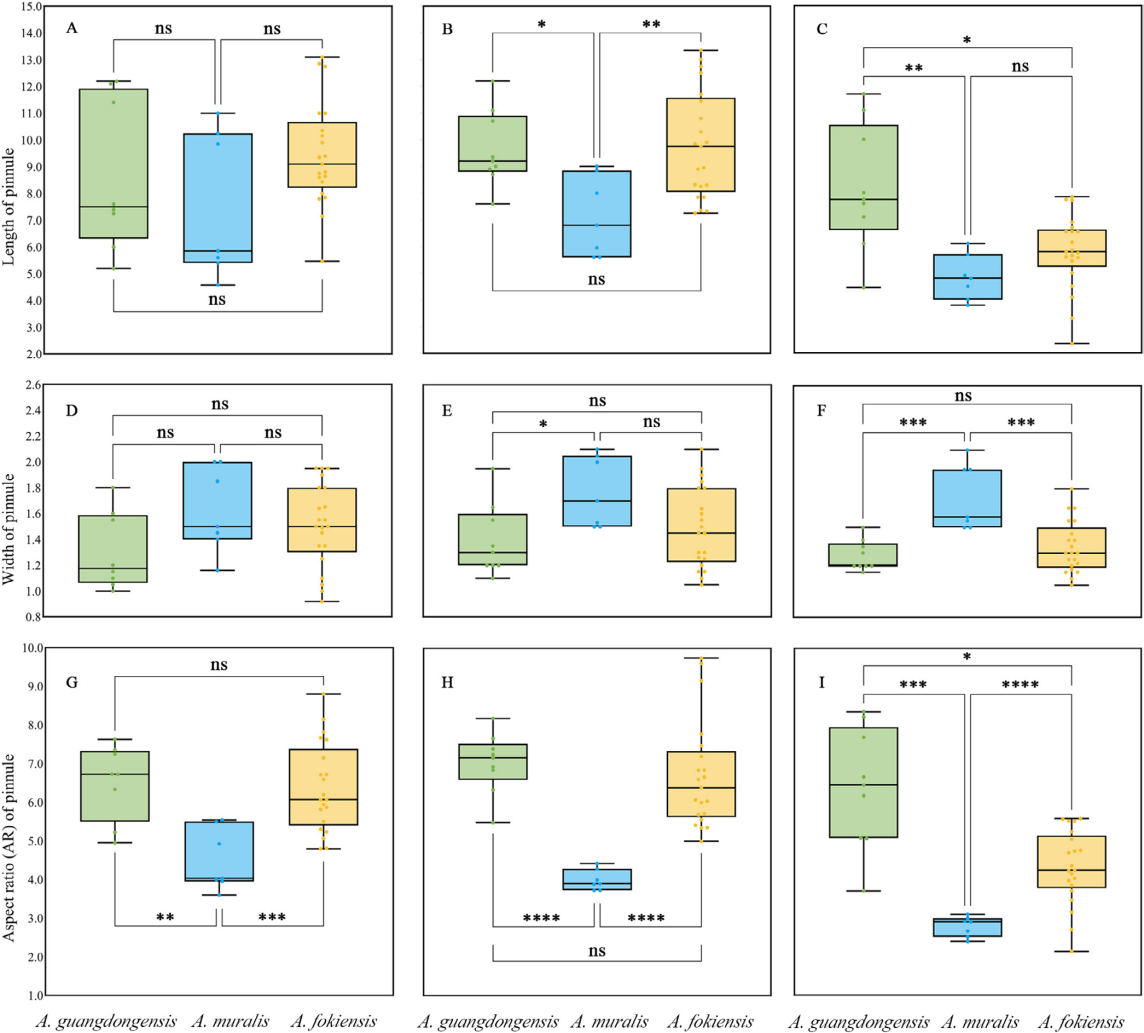
Significant difference analysis of length, width and aspect ratio (AR) between *A. guangdongensis* and the four most similar species (*, **, ***, and **** means significant at 0.01 < *p* ≤ 0.05, 0.001 < *p* ≤ 0.0l, *p* ≤ 0.001, *p* ≤ 0.0001; ns = not significant (*p* ≥ 0.05)). (A, D, G) Upper pinnule (B, E, H), Middle pinnae (C, F, I), Basal pinnule.

**Diagnosis**: *Angiopteris guangdongensis* is similar to both *A. fokiensis* (type locality: Fujian Province, China) and *A. muralis* (type locality: Guangdong Province, China), but it can be distinguished by the following diagnostic characters: (1) arborescent habit, 3–5 m tall (vs. 2–4 m in *A. fokiensis* and 2–3 m in *A. muralis* ); (2) robust stipes, 9 cm in diameter, densely scaly (vs. 6 cm, sparsely scaly in both species); (3) linear‐lanceolate pinnules (vs. lanceolate in *A. fokiensis* ; ovate, lanceolate, acuminate in *A. muralis* ); (4) pinnules densely scaly on the abaxial surface (vs. glabrous in *A. fokiensis* and sparsely scaly in *A. muralis* ); (5) higher basal pinnule AR (Figures 5, 6, 7, 8; Table 2).

**TABLE 2:** Morphological comparison with similar species.

Characters	*A. guangdongensis*	*A. muralis*	*A. fokiensis*
Frond	3–5 m	2–3 m	2–4 m
Stipe	Tuberculate or smooth	Tuberculate	Tuberculate or smooth
Rhizomes	Erect	Erect	Erect
Scales of the stipe	Dense	Sparse	Sparse
Laminae	Bipinnate	Bipinnate	Bipinnate
Pinnae	70–80 × 11–22 cm, pinnule 30–40 pairs	ca. 68 × 7–13 cm, pinnule 15–30 pairs	50–60 × 14–18 cm, pinnule 35–40 pairs
Spicules on the dorsal rachis	Absent	Present	Absent
Shape of pinnule	Linear‐lanceolate	Ovate, lanceolate, acuminate	Lanceolate
Abaxially scaly pinnules	Dense	Sparse	Glabrous
Veins	Veins present, not extending beyond the sori	Veins are obvious, extending nearly to the apices	Veins are obvious, extending nearly to the apices
Magin	Nearly entire	Regularly serrulate	Regularly crenulate
Sori	Sori 1.5–2 mm, with 7–16 sporangia	Sori 1–1.5 mm, with 12–16 sporangia	Sori ca. 1.5 mm, with 8–10 sporangia
Equatorial axis and polar axis of the spore	21.69 (19.63–24.08) μm × 23.15 (21.09–25.02) μm	20.03 (17.66–21.75) μm × 21.49 (19.02–23.68) μm	19.51 (18.73–20.84) μm × 22.2 (20.08–26.43) μm
References	/	(Ching 1959)	(Ching 1959; He and Christenhusz 2013)

**Description**: Plants are terrestrial. Fronds 3–5 m; rhizomes erect; stipes tuberculate or smooth, 9 cm in diameter, densely covered with fine, short, appressed scales; laminae bipinnate, with 3–6 pairs of pinnae; pinnae lanceolate, lower opposite‐alternate and upper alternate, linear‐oblong, widest at the middle, short‐stalked, 70–80 × 11–22 cm, with 30–40 pairs of pinnules; pinnules linear‐lanceolate, opposite or subopposite, 4.5–12 × 1–1.95 cm, densely scaly along the midrib abaxially, base cordate or rounded and nearly sessile, margins nearly entire, apices caudate; veins costate, free, < 1 mm apart, distinct abaxially but inconspicuous adaxially, simple or bifurcate, not extending beyond sori; false veins irregular, when present extending nearly to sori; pinnules papery, upper surface green, lower surface light green; when dry, rachises reddish‐brown, dorsal surface smooth and densely scaly, ventral surface with longitudinal grooves and narrow wings; sori ca. 1 mm from margin, 1.5–2 mm in diameter, with 7–16 sporangia; exospores with verrucate ornamentation, trilete.

**Distribution and ecology**: Known only from the type locality, where it grows on humid‐thermo ferralitic soils (pH 3.5–6) under light intensities of 696–1900 lx. It occurs at elevations of 149–165 m in areas receiving 1880–2650 mm of annual rainfall, with a mean annual temperature of 21.7°C. The habitat lies within a well‐preserved, pristine secondary evergreen broad‐leaved forest. Compared with its closely related species, *A. guangdongensis* exhibits a stable annual mean temperature, a narrower elevational range and stricter soil specificity (Table S2). These traits reflect unique adaptations in thermal tolerance, elevational preference and edaphic requirements, demonstrating its distinct niche differentiation as a lowland thermal specialist adapted to stable warm‐humid conditions.

**Etymology**: The specific epithet guangdongensis refers to Guangdong Province, China, the geographical origin of the type specimen. The species shows narrow endemism, being restricted to this region based on current distribution records.

**Vernacular name**: 广东观音座莲 (guang dong guan yin zuo lian).

**Conservation status**: *Angiopteris guangdongensis* is currently known only from Jiangmen, Guangdong Province, China, where it occurs in a nature reserve relatively unaffected by human disturbance. Approximately 40 mature individuals were recorded at the type locality. Based on current evidence and following International Union for Conservation of Nature (IUCN) Categories and Criteria (IUCN 2024), its restricted geographic range and small population size suggest that the species should be assessed as ‘Data Deficient (DD)’.

## 4 Discussion

The genus *Angiopteris* is broadly distributed across tropical and subtropical Asia, but species delimitation remains challenging due to pronounced morphological plasticity (Murdock 2008; Zhou and He 2025). This issue is especially evident in the *A. fokiensis* complex, where 14 previously described species were synonymised without thorough validation (Flora of Fujian 1991; Lin 2006; He 2009). Our field observations indicate that even protologue‐defined diagnostic traits (e.g., tuberculate stipes) exhibit extensive phenotypic variation, calling into question the reliability of traditional morphology‐based classifications. These findings highlight the need to integrate multidisciplinary approaches to resolve species boundaries in morphologically plastic groups.

Notably, pinnule morphology has proven to be more taxonomically informative than other characters (Lin 2006). Quantitative analyses of pinnule length, width and AR provide robust diagnostic criteria (Wang et al. 2021; Hong 2025). By conducting systematic morphometric measurements across different pinnule positions, our integrated approach distinguishes three taxa—*Angiopteris guangdongensis*, *A. muralis* and *A. fokiensis* —which form discrete morphological clusters (Figure 8). These well‐defined clusters offer strong preliminary evidence supporting the hypothesis that this complex harbours cryptic species.

Molecular phylogenetic analyses provide robust support for *Angiopteris guangdongensis* sp. nov. (BS = 100%, PP = 1.00) as a distinct evolutionary lineage, forming a well‐resolved sister clade to *A. fokiensis* (Figures 3 and 4). The substantial genetic divergence between *A. fokiensis* and *A. muralis* , together with diagnostic morphometric differences (Figures 3, 4, 5, 6, 7, 8; Table 2), further indicates that *A. muralis* may represent a cryptic species within the *A. fokiensis* complex.

Critically, plastome structure in *Angiopteris* is broadly conserved across the genus (Jiang 2020), yet interspecific divergence appears to be driven by variation at specific loci. Although *A. guangdongensis* shows strong intraspecific plastome conservation (Table S3), global sequence alignment analysis (Figure 2) revealed notable differences in the protein‐coding gene *ycf3* and the *trnI‐CAU–trnI‐CAU* spacer, which may contribute to plastome reorganisation (Frazão et al. 2023). These loci represent promising candidate molecular markers and may reflect functional adaptive divergence. We therefore propose the hypothesis that genetic differentiation in functional genes underlies the niche separation and evolutionary independence of these morphologically similar cryptic species.

Accordingly, *Angiopteris guangdongensis* is herein described as a new species, and the genomic data presented offer a critical foundation for advancing phylogenetic and taxonomic studies of the genus *Angiopteris*.

## Author Contributions

**Wu‐Feng Chen:** data curation (lead), formal analysis (lead), investigation (lead), methodology (lead), software (lead), validation (lead), visualization (lead), writing – original draft (lead), writing – review and editing (lead). **Wei‐Yue Sun:** resources (supporting), supervision (supporting), writing – original draft (supporting), writing – review and editing (supporting). **Li‐Jun Chen:** funding acquisition (equal), visualization (supporting). **Jiang‐Ping Shu:** funding acquisition (supporting), investigation (supporting), supervision (supporting), writing – review and editing (supporting). **Jun‐Jie Liang:** investigation (supporting), resources (supporting). **Yue‐Bing Zheng:** investigation (supporting), resources (supporting). **Yue‐Hong Yan:** conceptualization (lead), funding acquisition (equal), investigation (lead), methodology (supporting), project administration (lead), supervision (lead), writing – review and editing (equal).

## Conflicts of Interest

The authors declare no conflicts of interest.

## Supporting information


**Table S1:** The voucher information of complete plastid genome used in this study.

**Table S2:** Comparative analysis of climatic and environmental parameters for the habitats of *A. guangdongensis* , *A. muralis* and *A. fokiensis* .

**Table S3:** List of vouchers used in this study.

## Data Availability

The genome sequences generated in this study have been deposited in the GenBase database of the China National Center for Bioinformation (CNCB) (https://ngdc.cncb.ac.cn/genbase/). Accession numbers and corresponding voucher specimen information are provided in Table S3. Voucher specimens of the new species are deposited at NOCC and CSH.

## References

[ece372447-bib-0001] Andrews, S. 2010. “FastQC: A Quality Control Tool for High Throughput Sequence Data.” https://www.bioinformatics.babraham.ac.uk/projects/fastqc/.

[ece372447-bib-0002] Brudno, M. , S. Malde , A. Poliakov , et al. 2003. “Glocal Alignment: Finding Rearrangements During Alignment.” Bioinformatics 19: i54–i62. 10.1093/bioinformatics/btg1005. 12855437

[ece372447-bib-0003] Bui, Q. M. , H. A. Schmidt , O. Chernomor , et al. 2020. “IQ‐TREE 2: New Models and Efficient Methods for Phylogenetic Inference in the Genomic Era.” Molecular Biology and Evolution 37: 1530–1534. 10.1093/molbev/msaa015. 32011700 PMC7182206

[ece372447-bib-0004] Capella‐Gutiérrez, S. , J. M. Silla‐Martínez , and T. Gabaldón . 2009. “trimAl: A Tool for Automated Alignment Trimming in Large‐Scale Phylogenetic Analyses.” Bioinformatics 25, no. 15: 1972–1973. 10.1093/bioinformatics/btp348. 19505945 PMC2712344

[ece372447-bib-0005] Ching, R.‐C. 1959. Flora Reipublicae Popularis Sinicae. Science Press, 406 pp.

[ece372447-bib-0006] Flora of Fujian Editorial Committee, Fujian Provincial Commission of Science and Technology . 1991. Flora of Fujian. Fujian Science and Technology Press, 638 pp.

[ece372447-bib-0007] Frazão, A. , V. A. Thode , and L. G. Lohmann . 2023. “Comparative Chloroplast Genomics and Insights Into the Molecular Evolution of *Tanaecium* (Bignonieae, Bignoniaceae).” Scientific Reports 13: 12469. 10.1038/s41598-023-39403-z. 37528152 PMC10394017

[ece372447-bib-0008] Frazer, K. A. , L. Pachter , A. Poliakov , E. M. Rubin , and I. Dubchak . 2004. “VISTA: Computational Tools for Comparative Genomics.” Nucleic Acids Research 32: W273–W279. 10.1093/nar/gkh458. 15215394 PMC441596

[ece372447-bib-0009] GraphPad Software . 2023. “GraphPad Prism.” Version 10.1.2. Accessed May 12, 2025. https://www.graphpad.com.

[ece372447-bib-0010] Greiner, S. , P. Lehwark , and R. Bock . 2019. “OrganellarGenomeDRAW (OGDRAW) Version 1.3.1: Expanded Toolkit for the Graphical Visualization of Organellar Genomes.” Nucleic Acids Research 47: W59–W64. 10.1093/nar/gkz238. 30949694 PMC6602502

[ece372447-bib-0011] He, Z.‐R. 2009. Systematics of the Angiopteridaceae in China. Yunnan University, 99 pp.

[ece372447-bib-0012] He, Z.‐R. , and M. J. M. Christenhusz . 2013. “Marattiaceae.” In Flora of China. Vols. 2–3 (Pteridophytes), edited by Z.‐Y. Wu , P. H. Raven , and D.‐Y. Hong , 82–89. Science Press, Beijing & Missouri Botanical Garden Press.

[ece372447-bib-0013] Hieronymus, G. H. E. W. 1919. “Bemerkungen zur Kenntnis der Gattung *Angiopteris* Hoffm.” Hedwigia 61: 275–276.

[ece372447-bib-0014] Hoffmann, G. F. 1796. “Descriptiones et Icones Plantarum.” Commentationes Societatis Regiae Scientiarum Gottingensis 12: 29–30. http://resolver.sub.uni‐goettingen.de/purl?PPN35283028X_0012_1NS.

[ece372447-bib-0015] Hong, D. Y. 2025. “A Brief Discussion on Methodology in Taxonomy.” Biodiversity Science 33: 1–9. 10.17520/biods.2024541.

[ece372447-bib-0016] IUCN . 2024. Guidelines for Using the IUCN Red List Categories and Criteria. Version 16. Standards and Petitions Committee Accessed March 17, 2025. https://www.iucnredlist.org/documents/RedListGuidelines.pdf.

[ece372447-bib-0017] Jiang, Q.‐Y. 2020. Comparative Plastid Genomics and Phylogenetics Study of the “Molecular Living Fossil” Angiopteris (Marattiaceae). University of Chinese Academy of Sciences, 64 pp.

[ece372447-bib-0018] Jin, J.‐J. , W.‐B. Yu , J.‐B. Yang , et al. 2020. “GetOrganelle: A Fast and Versatile Toolkit for Accurate de Novo Assembly of Organelle Genomes.” Genome Biology 21: 241. 32912315 10.1186/s13059-020-02154-5 PMC7488116

[ece372447-bib-0019] Kalyaanamoorthy, S. , Q. M. Bui , T. K. F. Wong , A. Von Haeseler , and L. S. Jermiin . 2017. “ModelFinder: Fast Model Selection for Accurate Phylogenetic Estimates.” Nature Methods 14: 587–589. 10.1038/nmeth.4285. 28481363 PMC5453245

[ece372447-bib-0020] Katoh, K. , and D. M. Standley . 2013. “MAFFT Multiple Sequence Alignment Software Version 7: Improvements in Performance and Usability.” Molecular Biology and Evolution 30, no. 4: 772–780. 10.1093/molbev/mst010. 23329690 PMC3603318

[ece372447-bib-0021] Kaulfuss, G. F. 1824. Enumeratio Filicum Quas Im Itinere Circa Terram Legit Cl. Adalbertus de Chamisso Adiectis in Omnia Harum Plantarum Genera Permultasque Species Non Satis Cognitas vel Novas Animadversionibus… Cum Tabulis Aeneis Duabis. Carolus Cnobloch, 31 pp.

[ece372447-bib-0022] Kearse, M. , R. Moir , A. Wilson , et al. 2012. “Geneious Basic: An Integrated and Extendable Desktop Software Platform for the Organization and Analysis of Sequence Data.” Bioinformatics 28, no. 12: 1647–1649. 10.1093/bioinformatics/bts199. 22543367 PMC3371832

[ece372447-bib-0023] Lin, S.‐B. 2006. Taxonomic Treatment of the Genus Angiopteris Hoffm. in China. South China Agricultural University, 67 pp.

[ece372447-bib-0024] Murdock, A. G. 2008. “Phylogeny of Marattioid Ferns (Marattiaceae): Inferring a Root in the Absence of a Closely Related Outgroup.” American Journal of Botany 95, no. 5: 626–641. 21632388 10.3732/ajb.2007308

[ece372447-bib-0025] Qu, X.‐J. , M. J. Moore , D.‐Z. Li , and T.‐S. Yi . 2019. “PGA: A Software Package for Rapid, Accurate, and Flexible Batch Annotation of Plastomes.” Plant Methods 15: 50. 10.1186/s13007-019-0435-7. 31139240 PMC6528300

[ece372447-bib-0026] Ronquist, F. , J. Huelsenbeck , M. Teslenko , and J. Nylander . 2019. “MrBayes Version 3.2 Manual: Tutorials and Model Summaries.” Version 3.2. Accessed May 21, 2025. https://gensoft.pasteur.fr/docs/mrbayes/3.2.7/Manual_MrBayes_v3.2.pdf.

[ece372447-bib-0027] Ronquist, F. , M. Teslenko , P. Van Der Mark , et al. 2012. “MrBayes 3.2: Efficient Bayesian Phylogenetic Inference and Model Choice Across a Large Model Space.” Systematic Biology 61, no. 3: 539–542. 10.1093/sysbio/sys029. 22357727 PMC3329765

[ece372447-bib-0028] Wang, T. 2022. Phylogenetic Classification and Adaptive Evolution of Angiopteris in China. Southwest Forestry University, 140 pp.

[ece372447-bib-0029] Wang, T. , B. Xiao , E.‐D. Liu , et al. 2020. “Rediscovery of *Angiopteris tonkinensis* (Marattiaceae) After 100 Years, and Its Revision.” Phytokeys 161: 1–9. 10.3897/phytokeys.161.54912. 33005086 PMC7508941

[ece372447-bib-0030] Wang, T. , T. Yang , J.‐G. Zhang , et al. 2024. “ *Angiopteris nodosipetiolata* (Marattiaceae), a New Fern Species From Yunnan, China.” Phytokeys 241: 177–189. 10.3897/phytokeys.241.115175. 38721011 PMC11077261

[ece372447-bib-0031] Wang, T. , G.‐L. Zhang , Y.‐H. Yan , et al. 2021. “ *Angiopteris sugongii* (Marattiaceae), a New Diploid Species With Transitional Morphology Between *Angiopteris* (Sen. Str.) and *Archangiopteris* .” Phytotaxa 516, no. 3: 275–282. 10.11646/phytotaxa.516.3.6.

[ece372447-bib-0032] Wick, R. R. , M. B. Schultz , J. Zobel , and K. E. Holt . 2015. “Bandage: Interactive Visualization of de Novo Genome Assemblies.” Bioinformatics 31, no. 20: 3350–3352. 10.1093/bioinformatics/btv383. 26099265 PMC4595904

[ece372447-bib-0033] Xiang, C.‐Y. , F.‐L. Gao , I. Jakovlić , et al. 2023. “Using PhyloSuite for Molecular Phylogeny and Tree‐Based Analyses.” iMeta 2, no. 2: e87. 10.1002/imt2.87. 38868339 PMC10989932

[ece372447-bib-0034] Xie, J.‐M. , Y.‐R. Chen , G.‐J. Cai , R.‐L. Cai , Z. Hu , and H. Wang . 2023. “Tree Visualization by One Table (tvBOT): A Web Application for Visualizing, Modifying and Annotating Phylogenetic Trees.” Nucleic Acids Research 51: W587–W592. 10.1093/nar/gkad359. 37144476 PMC10320113

[ece372447-bib-0036] Zhang, D. , F.‐L. Gao , I. Jakovlić , et al. 2020. “PhyloSuite: An Integrated and Scalable Desktop Platform for Streamlined Molecular Sequence Data Management and Evolutionary Phylogenetics Studies.” Molecular Ecology Resources 20: 348–355. 10.1111/1755-0998.13096. 31599058

[ece372447-bib-0037] Zhao, J. , X.‐M. Zhou , S.‐L. Fang , et al. 2023. “Transcriptome‐Based Study on the Phylogeny and Hybridization of Marattialean Ferns (Marattiaceae).” Plants 2023, no. 12: 2237. 10.3390/plants12122237. PMC10301251 37375862

[ece372447-bib-0038] Zhou, X.‐M. , and Z.‐R. He . 2025. Systematic Taxonomy of Lycophytes and Ferns. Science Press, 340 pp.

