# Correction to “*Angiopteris guangdongensis* (Marattiaceae): A New Species From Guangdong, China”

**DOI:** 10.1002/ece3.72631

**Published:** 2026-01-29

Chen, W.‐F., Sun, W.‐Y., Chen, L.‐J., et al. 2025. “*Angiopteris guangdongensis* (Marattiaceae): A New Species From Guangdong, China.” *Ecology and Evolution* 15:e72447. https://doi.org/10.1002/ece3.72447.

In paragraph 2 of the “3.3 Taxonomic Treatment”, the text “Type: CHINA. Guangdong Province: Maoming City, Xinyi County” was incorrect. This should have read as follows: “Type: CHINA. Guangdong Province: Jiangmen City, Enping County”.

We apologize for this error.

