# Supplementary figures and images for: Angiopteris guangdongensis (Marattiaceae): A New Species From Guangdong, China
Source: Ecol Evol. 2025 Nov 9;15(11):e72447. doi: 10.1002/ece3.72447 (PMC12597604; doi:10.1002/ece3.72447)

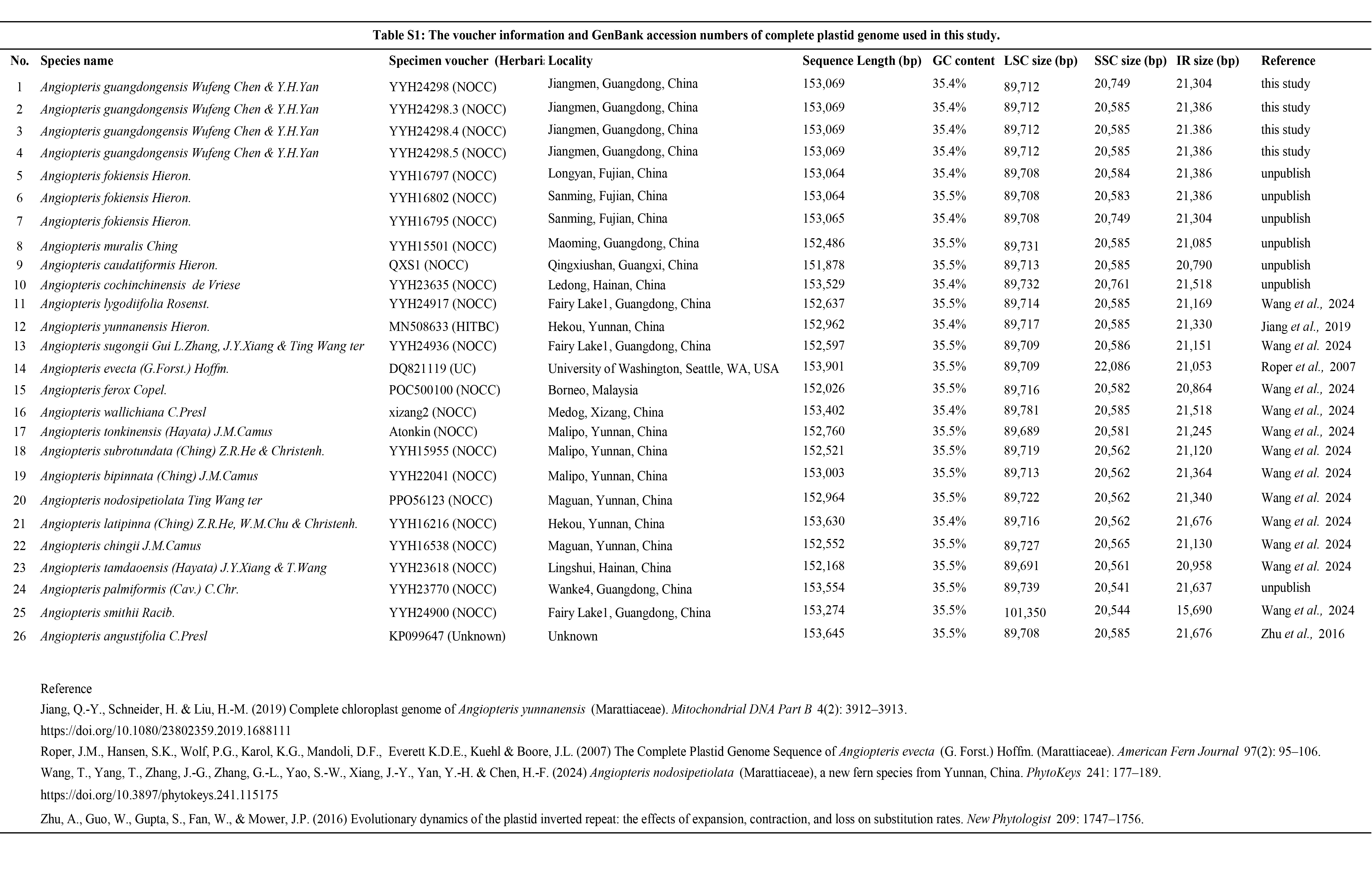

Supplement: Supplementary file 1 — Table S1: The voucher information of complete plastid genome used in this study. [file ECE3-15-e72447-s003.tif]

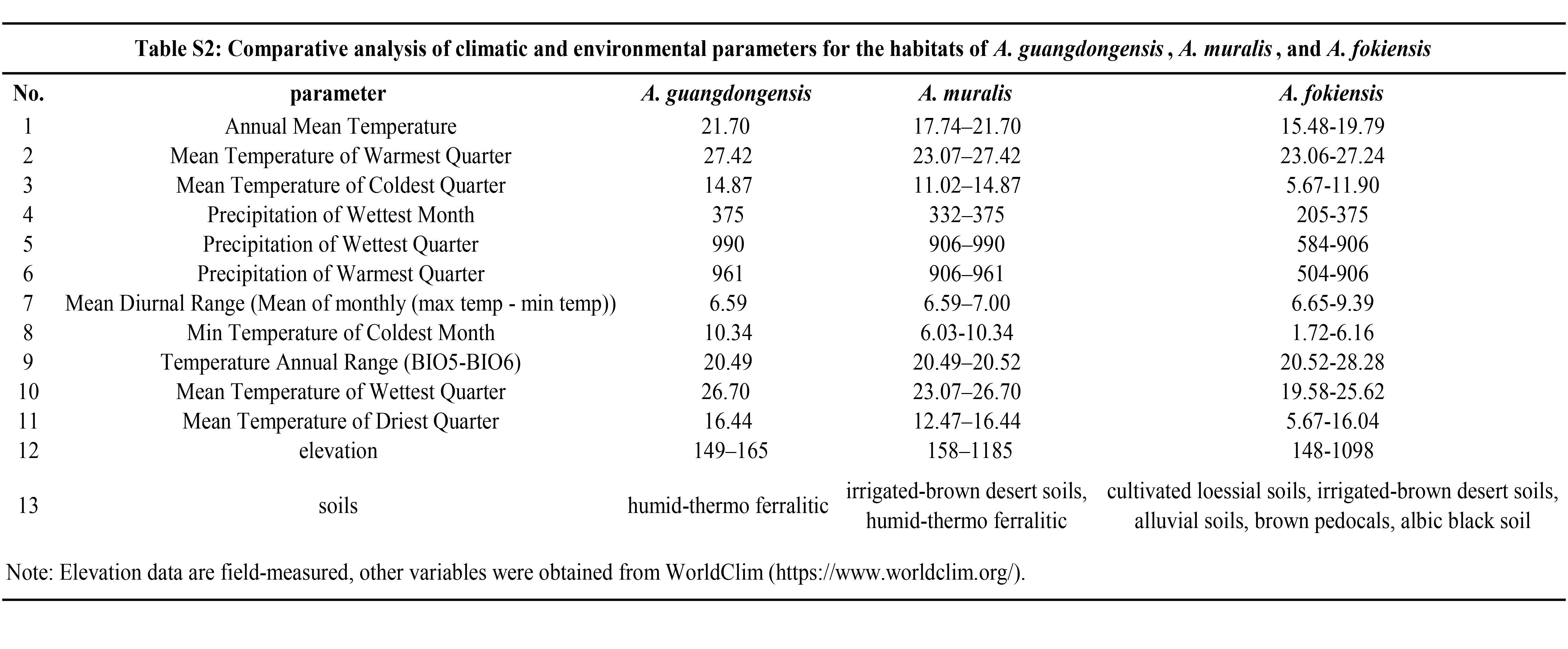

Supplement: Supplementary file 2 — Table S2: Comparative analysis of climatic and environmental parameters for the habitats of A. guangdongensis , A. muralis and A. fokiensis . [file ECE3-15-e72447-s002.tif]

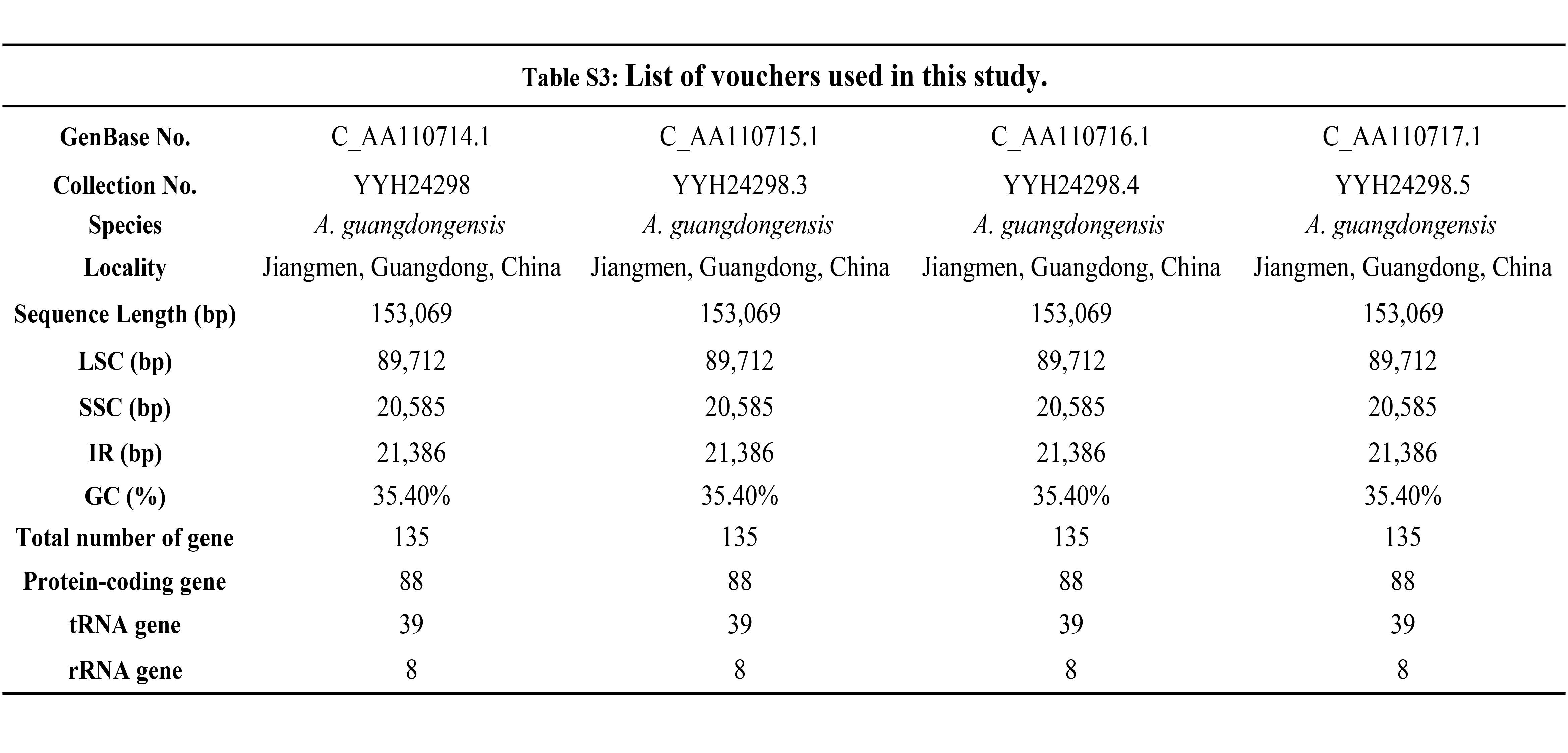

Supplement: Supplementary file 3 — Table S3: List of vouchers used in this study. [file ECE3-15-e72447-s001.tif]
